# Music Training, Working Memory, and Neural Oscillations: A Review

**DOI:** 10.3389/fpsyg.2020.00266

**Published:** 2020-02-21

**Authors:** Kate A. Yurgil, Miguel A. Velasquez, Jenna L. Winston, Noah B. Reichman, Paul J. Colombo

**Affiliations:** ^1^Department of Psychological Sciences, Loyola University, New Orleans, LA, United States; ^2^Department of Psychology, Tulane University, New Orleans, LA, United States; ^3^Brain Institute, Tulane University, New Orleans, LA, United States

**Keywords:** music training, working memory, neural oscillations, frequency bands, executive functions

## Abstract

This review focuses on reports that link music training to working memory and neural oscillations. Music training is increasingly associated with improvement in working memory, which is strongly related to both localized and distributed patterns of neural oscillations. Importantly, there is a small but growing number of reports of relationships between music training, working memory, and neural oscillations in adults. Taken together, these studies make important contributions to our understanding of the neural mechanisms that support effects of music training on behavioral measures of executive functions. In addition, they reveal gaps in our knowledge that hold promise for further investigation. The current review is divided into the main sections that follow: (1) discussion of behavioral measures of working memory, and effects of music training on working memory in adults; (2) relationships between music training and neural oscillations during temporal stages of working memory; (3) relationships between music training and working memory in children; (4) relationships between music training and working memory in older adults; and (5) effects of entrainment of neural oscillations on cognitive processing. We conclude that the study of neural oscillations is proving useful in elucidating the neural mechanisms of relationships between music training and the temporal stages of working memory. Moreover, a lifespan approach to these studies will likely reveal strategies to improve and maintain executive function during development and aging.

## Introduction

Music training engages some of our most complex cognitive abilities (Peretz and Zatorre, 2005; Zatorre and McGill, 2005) and induces brain plasticity in widely distributed cortical regions (Altenmüller and Schlaug, 2015). Musicianship requires selective and flexible attention, as well as inhibition of irrelevant auditory and visual stimuli. Musicians must readily manipulate stored information in accordance with complex hierarchies of rules and conventions. Reading musical notation requires strong spatial associations with percepts and symbols, and performing music requires effortful self-regulation and emotional expression.

Music training is emerging as an important model system for studying experience-dependent brain plasticity, and for the development of therapeutic interventions for healthy brain development and aging. Reports over the last few decades have firmly established that music training is associated with improvements on measures of executive functions, such as inhibitory control (Moreno and Farzan, 2015; D’Souza et al., 2018), working memory (Pallesen et al., 2010; Oechslin et al., 2013; Okhrei et al., 2016; Ding et al., 2018; D’Souza et al., 2018), and cognitive flexibility [see Okada and Slevc (2016) for a review], which coincide with structural and functional changes in brain regions implicated in these cognitive processes.

In studies using electroencephalography (EEG) and magnetoencephalography (MEG) to measure brain activity, inhibitory control is the most frequently reported executive function that is enhanced by music training, and it is frequently studied by measuring event-related potentials (ERPs), which coincide with discrete events. However, the relationship between music training and other components of executive function, such as working memory, has been studied less. In addition to ERPs, measurement of neural oscillations is a complementary method to study relationships between music training, working memory, and brain plasticity. In specific, the study of neural oscillations is well-suited for the study of events over periods of time from seconds to minutes, corresponding to the temporal stages of working memory. This review is focused on reports that link music training, working memory, and neural oscillations. The evidence that music training is associated with improvement in behavioral measures of working memory continues to increase. In addition, there is strong evidence linking behavioral measures of working memory to both localized and distributed patterns of neural oscillations (Tallon-Baudry et al., 1998; Jensen and Tesche, 2002; Kaiser et al., 2003, 2008, 2009; Herrmann et al., 2004; Grimault et al., 2009; Haegens et al., 2009; Moran et al., 2010; Van Dijk et al., 2010; Palva et al., 2011; Roux et al., 2012). And there is a small but growing number of reports of relationships between music training, working memory, and neural oscillations.

Taken together, we suggest that further study of neural oscillations will lead to significant progress in understanding the neural mechanisms that support effects of music training on behavioral measures of executive functions. The current review is divided into five main sections as follows: (1) Discussion of behavioral measures of working memory, and effects of music training on working memory in adults; (2) effects of music training on neural oscillations during distinct temporal stages of working memory; (3) relationships between music training and working memory in children; (4) relationships between music training and working memory in older adults; and (5) effects of entrainment of neural oscillations on cognitive processing.

### Behavioral Measures of Working Memory in Adults

There is increasing interest in the effects of music training on enhancement of general cognitive abilities and intelligence (Moreno et al., 2011; Schellenberg, 2011; Schellenberg and Weiss, 2013; Zuk et al., 2014; Costa-Giomi, 2015), and evidence suggests that working memory is an important component of the cognitive benefits derived from music training (Bergman Nutley et al., 2014; Suárez et al., 2015). For the purposes of this review, we define working memory as time- and capacity-limited storage of task-relevant information, which generally requires one or more of the following operations: mental manipulation, flexible use, or inhibition of distractors. Although working memory is sometimes equated with short-term memory, we consider it distinct for two reasons. First, working memory, as defined above, is distinct from short-term storage in the general requirement of mental manipulations of encoded information, or the inhibition of goal-irrelevant stimuli. Second, working memory requires integrity of medial temporal lobe regions, whereas short-term memory does not.

Working memory is often studied using behavioral tasks that implement a combination of stored information, cognitive manipulation, and interference. These tasks include the *N*-back (Pallesen et al., 2010; Oechslin et al., 2013; Ding et al., 2018), backward digit span (George and Coch, 2011; Zuk et al., 2014; Clayton et al., 2016), reading span (Franklin et al., 2008; D’Souza et al., 2018), and operation span tasks (Franklin et al., 2008; D’Souza et al., 2018). For the *N*-back task, a sequence of visual or auditory stimuli is presented, and the participant maintains the information while deciding whether each subsequent stimulus item matches the stimulus that came *N* letters previously (Owen et al., 2005). For the backward digit span, participants are presented with a series of digits, then asked to report the sequence of digits in reverse order (Hester et al., 2004). Both the *N*-back and the backward digit span require participants to maintain information in memory and manipulate the information in a certain order. The *N*-back may be a more demanding task as the participant must simultaneously keep track of current stimuli and determine whether or not they match the stimulus *N* turns back. For the reading span task, *N* number of sentences are presented one sentence at a time. As *N* increases, so does memory load required to perform the task. For *N* = 2, two sentences are presented sequentially, and after each sentence is presented, the participant writes the sentence verbatim, and the last word of each sentence in order (Daneman and Carpenter, 1980). Finally, the operation span task requires participants to memorize a sequence of unrelated words while simultaneously performing a series of math operations. After all of the operation-word strings are presented, the participant writes all of the words that were displayed in the order of presentation (Turner and Engle, 1989). Therefore, both the reading span and the operation span require participants to hold information while working on a secondary task, which may cause interference. As noted above, each of these tasks fulfill the criteria of maintenance and manipulation of information, which may occur with differing levels of interference (Aben et al., 2012). Working memory performance on these and related tasks will be discussed in the paragraphs that follow in relation to music training and neural oscillations.

## Relationship Between Music Training and Working Memory in Adults

Musicians reportedly outperform non-musicians on a variety of working memory tasks. For example, musicians showed better working memory capacity and duration than non-musicians using forward tonal discrimination tasks, whereas under atonal conditions, musicians outperformed non-musicians on working memory capacity, but not duration (Ding et al., 2018). Thus, musicians held more items in memory for both tonal and atonal auditory stimuli, but retained the items longer than non-musicians only for tonal stimuli. Furthermore, musicians have been reported to outperform non-musicians on *N*-back tasks using both auditory (Pallesen et al., 2010; Ding et al., 2018) and visual (Oechslin et al., 2013; Okhrei et al., 2016) stimuli. Therefore, the benefits of music training for working memory are not necessarily limited to the auditory domain. Musicians have enhanced verbal working memory compared to non-musicians, as measured by both reading and operation span tasks (Franklin et al., 2008). However, this advantage may not extend to the visuospatial domain (Hansen et al., 2013), as no significant differences were reported between musicians and non-musicians on the Colorado Assessment for visual working memory (Strait et al., 2014), a computerized visual working memory task (Okhrei et al., 2016), and a spatial span task (Hansen et al., 2013). Discrepant reports of effects of musicianship are most likely due to the type of information to be retained, and less likely due to the age of participants, the sample size, or the types of tasks used. Slevc et al. (2016) reported that music training predicts individual differences in working memory, but that effect is not as strongly related to inhibitory control, and unrelated to cognitive flexibility. Using a similar approach to study the relationships between music training and executive functions, Okada and Slevc (2018) used a test battery consisting of tasks related to three subcomponents of executive functions: working memory, inhibitory control, and cognitive flexibility. They reported a positive correlation between individual variation in music training and working memory updating, but no relationships between music training and inhibitory control or shifting. In these contexts, inhibitory control refers to one’s ability to regulate behavior, attention, and thoughts – especially in the face of conflicting information, or when doing so requires overriding a prepotent response. Cognitive flexibility refers to one’s ability to successfully switch between task demands or mental sets (Slevc et al., 2016; Okada and Slevc, 2018). Relationships between music training and inhibitory control and cognitive flexibility have been reviewed in other sources and will not be taken up here (Moreno and Farzan, 2015; Okada and Slevc, 2016). It should be noted, however, that understanding the relationships between music training and behavioral measures of executive functions will progress more rapidly if a reliable battery of tests of executive functions is used consistently among investigators. For example, the NIH Toolbox cognitive measures include measures of executive function and have the added benefit of normative data.

### Neural Oscillations and Working Memory in Adults

Neural oscillations are believed to represent synchronous changes in excitability in networks of cortical cells (Fries, 2005; Klimesch et al., 2007), and they are well-suited for the study of cognitive processes that occur over longer durations of time than a single event. This applies particularly to the study of the duration and capacity of the maintenance phase of working memory. Compared to intracranial recording techniques, EEG is a non-invasive and relatively inexpensive tool for measuring patterns of neuronal activity synchronized at the population level that may accompany sensory and cognitive processing. Electrical brain activity may be analyzed in the time-domain as ERPs, which are time-locked averages of EEG to a repeated stimulus or response event (see Glossary). While there is abundant research related to ERPs and working memory (Drew et al., 2006; Yurgil and Golob, 2013; Pinal et al., 2015; Getzmann et al., 2018), the current review focuses on spectral (time-frequency) methods as a complementary approach to extracting ongoing oscillatory features that correspond to cognitive operations, such as the encoding or short-term storage of information. Various time-frequency analyses can be used to determine EEG magnitude or power within a given frequency band as well as the degree of coherence between different cortical regions (see Glossary for description of different frequency bands and spectral measures). Although time-frequency analyses are typically more computationally intensive than time-domain ERP methods, characterizing cortical oscillations and their synchronization is advantageous in examining the brain’s distributed processes. In addition, there is strong evidence that different oscillations are supported by different functional networks that underlie sensory and cognitive processes, including working memory.

**Glossary:** 

Amplitude	Magnitude of an oscillatory signal, measured in microvolts
Coherence	A measure of oscillatory amplitude or phase consistency between a pair of electrodes
Cross-frequency coupling (CFC)	Interaction between two frequency bands such that one oscillation modulates the amplitude or phase of a second oscillation
Electroencephalography (EEG)	Non-invasive technique with millisecond temporal resolution used to measure changes in electrical cortical activity that accompanies sensory and cognitive processing
Event-related desynchronization (ERD)/synchronization (ERS)	Event-related difference in frequency band power relative to baseline; ERD reflects a decrease in post-stimulus EEG power, whereas ERS reflects an increase
Event-related potential (ERP)	Averaged EEG waveform time-locked to a stimulus or response event
Frequency band	Range of frequencies used in EEG analysis, denoted by different Greek letters: delta (1–4 Hz), theta (4–8 Hz), alpha (8–12 Hz), beta (12–30 Hz), and gamma (>30 Hz)
Neural oscillation	Rhythmic fluctuation in excitability of neural assemblies
Power	Squared amplitude of EEG signal within a frequency band
Phase	The position of an oscillation at a given point in time, measured in radians (0–2π) or degrees (0–360)
Phase synchrony	A measure of the relationship between the phases of two oscillations, irrespective of amplitude
Transcranial magnetic stimulation (TMS)	Non-invasive neuromodulatory technique in which magnetic pulses are used to stimulate a targeted brain region
Transcranial alternating current stimulation (tACS)	Non-invasive neuromodulatory technique that delivers oscillating current at a desired frequency

There is ample evidence that neural oscillations are important for memory formation. This was first demonstrated in studies of long-term potentiation (LTP) and long-term depression (LTD), which are neuronal models for memory formation involving biphasic changes in neuronal responses to synaptic inputs. Huerta and Lisman (1995) reported that a single burst of stimulation at the peak of theta phase induced LTP, whereas stimulation at the trough of theta induced LTD. The role of hippocampal theta has been investigated extensively in rodent models of memory formation (Buzsáki et al., 1994), and extended to interactions between the hippocampus and prefrontal cortex in humans (Anderson et al., 2010). Theta-band activity is also implicated in “chunking” of perceptual auditory information, which may contribute to hierarchical control representations necessary for complex, flexible behavior (Kikumoto and Mayr, 2018; Teng et al., 2018). In addition to theta, other neural oscillations, including alpha and gamma, are implicated in memory formation and maintenance. Indeed, Roux and Uhlhaas (2014) attributed distinct functional roles of alpha, theta, and gamma neural oscillations in working memory. In specific, they proposed that activity in the gamma-band subserves maintenance of working memory, whereas alpha-band oscillations may inhibit task-irrelevant information, and theta-band oscillations are important for temporal order of items in working memory. While significant advances have been made in understanding the roles of neural oscillations in working memory, less is known about the relationships between music training, neural oscillations, and their functional networks.

In this review, we offer a framework for examining oscillations related to different working memory components, and how music training may influence these underlying mechanisms. Specifically, we propose that oscillations may be used to: (1) dissociate temporal components of working memory, that is to distinguish between processing stages of encoding, maintenance, and retrieval; (2) investigate working memory processing demands, such as changes in memory load and inhibition; (3) measure short and long-range synchronous activity to examine the relative contributions of distributed brain regions involved during working memory tasks; and (4) examine the degree to which music training influences oscillatory activity during working memory.

#### Music Training, Neural Oscillations, and Working Memory Maintenance

Stimulus-related oscillations may fluctuate over time and are therefore advantageous when examining neural activity over memory delays. There is considerable evidence that working memory maintenance, or the process of sustaining information in the absence of sensory input, is associated with enhanced activity or coherence in theta (4–8 Hz; Jensen and Tesche, 2002; Moran et al., 2010), alpha (8–12 Hz; Herrmann et al., 2004; Grimault et al., 2009; Haegens et al., 2009; Van Dijk et al., 2010), beta (12–30 Hz) in non-human primates (Tallon-Baudry et al., 2004) and humans (Palva et al., 2011), and gamma (>30 Hz; Tallon-Baudry et al., 1998; Kaiser et al., 2003, 2008, 2009; Palva et al., 2011; Roux et al., 2012) frequency bands [see Roux and Uhlhaas (2014) for extensive review of theta, alpha, and gamma]. See Table 1 for a summary of results.

**TABLE 1:** This table shows the results of studies on young adults, working memory, and oscillatory activity.

**Study**	**Task**	**EEG measure**	**FB**	**Behavioral results**	**Oscillatory results**			
					**Encoding**	**Maintenance**	**Retrieval**	**Load**
Van Dijk et al., 2010	aDMS	Power	α	–	↑**α** (*T*)	↑**α** (*T*)	–	–
Grimault et al., 2009	vSTM	Power	α	–	–	↑**α**	–	↑Load = ↑**α** (*F, P*)
Haegens et al., 2009	DMS	Power	αβγ	73.7% correct responses	↑**γ** (contralateral, somatosensory) ↑**α** (contralateral, somatosensory) ↓**β** (contralateral, somatosensory)	↑**γ** (*bilateral SII*) ↑**α** (*O*)	–	–
Herrmann et al., 2004	vDMS	Power	α	–	–	↑**α** (*P*)	–	–
Jensen and Tesche, 2002	Sternberg	Power	θ	↑ Load = ↑ RT	–	↑Load = ↑θ(*F*) ↑Load = ↓**θ(***C, P*)	–	↑Load = ↑**θ**(*F*) ↑Load = ↓**θ**(*C, P*)
Kaiser et al., 2003	DMS	Power, coherence	γ	–	–	↑**γ** (*LT*) ↑**γ** (Coh. *LT - F*)	–	–
Meltzer et al., 2007	Sternberg		θα	↑ Load = ↑ RT	–		–	↑Load = ↑(θ(*FM*)
Moran et al., 2010	vDMS	Frequency	θ	Items remembered: 1.5 - 5	–	↑**θ**(*F, P*)	–	↑Load = ↑**θ**(*F, P*)
Palva et al., 2011	vDMS	Power	βγ	↑Load = ↓Accuracy	–	↑Load = ↑γ (O) ↑Load = ↑**β** (*O*)	–	↑Load = ↑**γ** (*O*) ↑Load = ↑**β** (*O*)
Roux et al., 2012	vDMS	Power	αγ	No difference in RT.	–	↑**γ**(*P*)	–	↑Load = ↑**γ**(*P*)
Samuel et al., 2018	vDMS	Frequency	α	–	↓**α** (Freq.)	↑**α** (Freq.)	↑**α** (Freq.)	–
Tallon-Baudry et al., 2004	vDMS	Phase synchrony	β	–	–	↑**β** (*P*)	–	–

*DMS, delay match-to-sample task; aDMS, auditory DMS; vDMS, visual DMS; F, frontal; C, central; T, temporal; P, parietal; O, occipital; M, medial; L, left; R, right. SII, supplementary somatosensory; RT, reaction time. Frequency bands: δ, delta (1–4Hz); θ, theta (4–8 Hz); α, alpha (8–12 Hz); β, beta (12–30 Hz); γ, gamma (>30 Hz).*

In addition, activity within each frequency band has been shown to vary with working memory load, or the number of items maintained over a brief delay (Table 1). High load conditions – that is, maintaining more information within working memory – are associated with increased power in alpha (Grimault et al., 2009; Palva et al., 2011; Samuel et al., 2018), beta (Palva et al., 2011), and gamma ranges (Howard et al., 2003; Palva et al., 2011). Increased load during memory delays is also associated with increased peak theta frequency (Moran et al., 2010), however findings on theta power and working memory load are mixed. Some studies show increased theta power with memory load over midline frontal (Jensen and Tesche, 2002; Meltzer et al., 2007) and other dispersed brain regions (Raghavachari et al., 2001, 2006), while others show decreased power with load over lateral frontal regions (Meltzer et al., 2007; van Vugt et al., 2010; Brzezicka et al., 2019).

While less studied, music training-related differences in working memory maintenance have been shown within the theta and beta ranges (Table 2). For example, music training may enhance theta-related activity during delay periods, affecting subsequent memory processing. Cheung et al. (2017) reported that musicians exhibited increased intra-hemispheric theta coherence during verbal memory encoding. Coherence is a measure of consistency within a given frequency band in amplitude or phase between different brain areas, thus higher coherence indicates greater synchrony of activity across regions. Higher theta coherence in musicians also correlated with subsequent memory performance (Cheung et al., 2017). Likewise, compared to non-musicians, musicians show increased left hemispheric theta coherence when judging the semantic relatedness of a new stimulus to that of previously learned information (Dittinger et al., 2017). Although not working memory maintenance *per se*, these tasks required sustained activation of memory representations for later processing; thus, music training may enhance theta coherence during tasks that require maintaining stimulus representations over time. Increased theta coherence may support connections between brain areas important for memory processing, including medial temporal lobe and prefrontal cortex (Jones and Wilson, 2005; Anderson et al., 2010). In addition, Hsu et al. (2017) compared beta activity in musicians and non-musicians during an auditory *N*-back task. Enhanced beta activity has been shown to facilitate maintenance of information over delays in non-human primates (Tallon-Baudry et al., 2004) and humans (Lundqvist et al., 2016), and is predictive of individual visual working memory capacity (Palva et al., 2011). Group differences in beta activity indicated a processing advantage for musicians over non-musicians. As these differences were observed during the first 30 s of the 0-back condition, music training may promote more efficient encoding and maintenance of information within working memory, in the absence of interference or distraction.

**TABLE 2:** This table summarizes the findings of the effects of music training on working memory and oscillatory activity.

**Study**	**Task**	**FB**	**EEG measure**	**Behavioral results**	**Oscillatory results**	
					**Active recording**	**Resting state**
Bhattacharya et al., 2001	Shepard–Metzler task of mental rotation	γ	Coherence	–	**γ** (*Overall*): M > NM **γ** (*L-hemi*): M > NM **γ** (*R-hemi*): M < NM	*–*
Boutorabi and Sheikhani, 2018	Spatial working memory	δθαβγ	Coherence	–	**δ** (*T, C, P*): M < NM **θ** (*F,C,P*): M < NM **α** (*C,P*): M < NM **β** (*F, T, C, P*): M < NM **γ** (*C*): M < NM **γ** (*F,T*): M > NM	–
Cheung et al., 2017	- HKLLT (verbal memory) - Visual reproduction (visual memory)	θ	Coherence	Verbal memory: M > NM Visual memory: M = NM	During encoding: **θ** (*long-range, intrahemi*.): M > NM	**θ**: M = NM
Dittinger et al., 2017	- Matching task - Semantic task - Backward digit span	θ	Coherence	Semantic task: M > NM BDS: M = NM	**θ** (*L-hemi*): M > NM ↑ Mus. Experience = ↑**θ** (*L dorsal pathways*)	–
Hsu et al., 2017	*N*-back (tonal)	β	Feature analysis	–	**β**: Response Speed: M > NM Response Intensity: M > NM Response Power: M > NM	–
Klein et al., 2016	Visual Sternberg	θαβ	Covariance mapping analysis	Correct responses: M = NM RT: M = NM	**α** (*F,P*): Mus prestimulus activity neg. correlates with RT.	**All FB:** M = NM

*M, musicians; NM, non-musicians; FB, frequency band; F, frontal; C, central; T, temporal; P, parietal; O, occipital; M, medial; L, left; R, right; RT, reaction time; BDS, backward digit span.*

There is also evidence that music training alters oscillatory coherence between different brain areas that are active during spatial working memory tasks (Table 2). Bhattacharya et al. (2001) examined the effects of music training on gamma coherence during mental rotation, a task that requires participants to discriminate between a 3D object and its rotated mirror image (Shepard and Metzler, 1971). Gamma coherence between frontal and right parietal cortex increased during mental rotation for all participants; however, musicians showed higher coherence overall and greater phase synchrony in left hemisphere compared to non-musicians. Consistent with visuospatial behavioral findings discussed above (Hansen et al., 2013; Strait et al., 2014; Okhrei et al., 2016), there were no behavioral group differences in mental rotation. However, differences in gamma synchrony and hemispheric recruitment suggest music training alters activity within functional networks that support spatial working memory processes, including parietal and prefrontal cortices (Alivisatos, 1992; Tagaris et al., 1996; Alivisatos and Petrides, 1997). In a more recent study, musicians demonstrated enhanced gamma coherence between frontal and temporal regions during a spatial working memory task, but reduced coherence across all other frequency bands compared to non-musicians (Boutorabi and Sheikhani, 2018). Enhanced coherence was observed across the entire trial, and thus not strictly limited to working memory maintenance. However, it appears that enhanced coherence observed in musicians mediates cortical interactions important for working memory.

#### Music Training, Neural Oscillations, and Working Memory Encoding and Retrieval

Fluctuations in oscillatory activity may drive not only the active maintenance of items over brief delays, but the encoding and retrieval of such items. In a visual delayed match to sample paradigm, higher memory loads were associated with decreases in alpha frequency and power during encoding, and increases in alpha frequency during retrieval (Samuel et al., 2018). In another study by Myers et al. (2014), pre-stimulus fluctuations in alpha power predicted accuracy during working memory retrieval; specifically, alpha event-related desynchronization (ERD; reduced power) was correlated with better memory performance.

Music training may further modulate pre-stimulus alpha activity, consequently affecting working memory retrieval (Table 2). In a study by Klein et al. (2016), pre-stimulus alpha activity over anterior–posterior regions was negatively associated with reaction time during a visual Sternberg task for musicians compared to non-musicians. According to findings from functional imaging studies, working memory recruits fronto-parietal brain areas (Wager and Smith, 2003; Owen et al., 2005), thus reduced anterior–posterior alpha activity may facilitate processing task-relevant information in musicians compared to non-musicians (Klein et al., 2016). Group differences in pre-stimulus alpha activity may reflect experience-dependent engagement of different neural networks involved in working memory.

Desynchronized oscillatory activity has been observed in other learning and memory paradigms. In a study by Silva et al. (2018), non-musicians learned to identify different melodic intervals, and were tested after an initial training session (baseline), and after 5 days of at-home training sessions (post-training). Improved accuracy and reaction time to learned intervals was accompanied by reductions in alpha, beta, and gamma activity from baseline to post-training. Taken together, these findings suggest that changes in oscillatory activity and their functional networks may reflect individual variability during encoding, maintenance, and retrieval of items within working memory.

#### Music Training, Neural Oscillations, and Distractor Inhibition

It is important to note that the majority of findings discussed above were derived from working memory tasks that probe temporary storage of information in the absence of processing task-irrelevant information. However, given that working memory is a capacity-limited resource, active inhibition of task-irrelevant information is an important and necessary executive function. Neural oscillations are a useful tool in dissociating between storage and processing components of working memory and their underlying functional networks.

There is substantial evidence that alpha oscillations, first observed by Berger (1929), are involved in attentional suppression of task-irrelevant information. According to the alpha inhibitory hypothesis, alpha desynchronization (reduced alpha power) is associated with active information processing while alpha synchronization (increased alpha power) is associated with active inhibition of task-irrelevant information [see Klimesch et al. (2007) and Foxe and Snyder (2011) for reviews].

Using a modified Sternberg task with weak and strong distracters, Bonnefond and Jensen (2012) found alpha activity increased with anticipation of strong distracters and was associated with faster reaction time to memory probes. Likewise, Sauseng et al. (2009) found increased alpha activity over posterior brain areas associated with distractor suppression, while maintaining relevant items was associated with increased theta-gamma interactions. Thus, high alpha activity facilitates inhibition of distracters during working memory. Furthermore, asymmetries in alpha power have been used to dissociate target selection vs. distractor inhibition functions within working memory (Schneider et al., 2019).

In tasks that require distractor suppression, individual differences in alpha activity may underlie behavioral performance during working memory tasks. Alpha activity has been shown to vary with working memory capacity, defined as the ability to actively maintain items in working memory in the face of distraction (Engle et al., 1999). Dong et al. (2015) found that independent of task difficulty, individuals with low working memory capacity exhibit greater alpha ERD (reduced alpha power), which may reflect the involvement of additional neural resources including those that are irrelevant to the task at hand.

To our knowledge, no literature exists on whether music training modulates oscillatory activity (alpha or otherwise) related to distractor inhibition. However, behavioral and ERP studies indicate better or more efficient inhibitory processing in musicians compared to non-musicians [see Moreno and Farzan (2015) for review]. While ERPs are useful in examining inhibition that is time-locked to a particular distracter event, measuring changes in oscillatory activity and coherence over longer periods of time may be useful to determine whether music training modulates distracter inhibition and associated functional networks over longer delays, as in working memory maintenance.

#### Music Training and Cross-Frequency Coupling During Working Memory

Neurons that oscillate at different frequencies may interact with each other, forming nested hierarchies in a time-dependent manner spanning multiple brain regions. Cross-frequency coupling, or the interaction of different frequency bands, is a useful tool in examining underlying generators and functional connectivity related to working memory.

It has been well established that working memory is associated with cross-frequency coupling between theta and gamma oscillations [Lisman and Jensen (2013) for review]. Studies show that the number of items maintained in working memory is related to the number of gamma rhythms nested within one theta cycle (Lisman and Idiart, 1995; Jensen and Lisman, 1998). This nesting predicts behavioral performance on working memory tasks, including response time (Axmacher et al., 2010) and individual differences in span (Kamiński et al., 2011). Theta-gamma coupling during working memory has been associated with activity in fronto-hippocampal networks in both rodents (Belluscio et al., 2012) and humans (Axmacher et al., 2010). Furthermore, modulation of theta-gamma rhythms in prefrontal cortex improves working memory performance in adults (Alekseichuk et al., 2016).

Besides theta–gamma interactions, other cross-frequency couplings have been shown to support working memory processes. In monkeys, alpha–gamma coupling in the fronto-parietal network is sensitive to changes in working memory load (Pinotsis et al., 2019). In humans, interactions between visual and fronto-parietal theta and alpha/gamma, and between alpha and beta/gamma oscillations are enhanced during working memory maintenance, and reflect interactions between sensory and fronto-parietal networks (Siebenhühner et al., 2016). Additionally, cross-frequency coupling increased with working memory load and predicted individual differences in working memory capacity.

Previous sections of this review discussed differences in activity within frequency bands related to musical expertise. However, to our knowledge, there are no reports of whether cross-frequency coupling during working memory varies as a function of music training. While music training-dependent interactions between frequency bands remain to be investigated, we provided evidence that music training may alter neural connectivity in functional networks underlying working memory. During spatial working memory tasks, musicians show enhanced gamma coherence in fronto-parietal (Bhattacharya et al., 2001) and fronto-temporal (Boutorabi and Sheikhani, 2018) networks. During verbal memory tasks, musicians show enhanced intra-hemispheric theta coherence (Cheung et al., 2017; Dittinger et al., 2017). Group differences in spectral coherence complement fMRI findings that musicians engage different but overlapping brain regions during verbal vs. tonal working memory tasks, while non-musicians recruit the same regions regardless of task (Schulze et al., 2011). Thus, musicians may rely on stronger or alternative functional networks when engaged in different working memory tasks. These findings may be supported by future investigations of the effects of music training on cross-frequency coupling during working memory.

## Relationships Between Music Training and Working Memory in Children

Several authors have reported that development of working memory may serve as a mechanism for emergence of cognitive abilities and other developmental outcomes (see Kraus et al., 2012). For example, there are reports of associations between working memory performance, music training or aptitude, and developmental outcomes such as neural encoding of speech (Strait et al., 2011, 2012; Christiner and Reiterer, 2018; Ireland et al., 2018), as well as cognitive skills related to reading abilities (Banai and Ahissar, 2013; Degé et al., 2015).

Behavioral investigations of children have shown that music training and musical aptitude are associated with enhanced auditory working memory. For example, in a longitudinal study of 6–8-year-old children, half of the sample was randomly assigned to biweekly keyboard training for 6 weeks, while the other half received no training. Afterward, only the training group demonstrated a significant improvement in working memory capacity, measured with the backward digit span task (Guo et al., 2018). Advantages in working memory capacity have also been observed in school-aged children who received at least 6 months of music lessons (Degé and Schwarzer, 2017), as well as pre-schoolers who received 1 year of violin instruction (Fujioka et al., 2006). Bergman Nutley et al. (2014) measured working memory capacity every 2 years on two or three occasions among participants aged 6–25 years. They reported that musical practice was associated with better working memory capacity at each timepoint, and the increase was proportional to the hours of weekly musical practice, suggesting a dose–response relation between musical practice and working memory capacity.

The findings of cross-sectional studies of musical training and working memory in children are mixed. One report showed a musician advantage for auditory but not visual working memory (Strait et al., 2012), while other reports showed no effect of music training on working memory (Banai and Ahissar, 2013; Sachs et al., 2017). It should be noted, however, that while Sachs et al. (2017) reported no effect of music training on working memory, they did find differences between children with music training and those without training in measures of neural activity in brain regions associated with cognitive processes. In addition, cross-sectional studies have examined the relationship between musical aptitude and working memory capacity. Such investigations have shown that higher working memory capacity is associated with better scores on rhythmic (Strait et al., 2011; Degé et al., 2015; Ireland et al., 2018) and tonal (Christiner and Reiterer, 2018) subtests of musical aptitude in children as young as 5 years.

One issue in studies of relationships between musical training and cognitive outcomes is the direction of causality. For example, those who are predisposed to succeed in music may also be predisposed to demonstrate enhanced cognitive abilities relative to age-matched peers (see Schellenberg, 2011). Alternatively, music training may cause enhancement of cognitive abilities. Longitudinal studies in which participants were randomly assigned to music training or control conditions clearly indicate a causal effect of training on working memory capacity (Fujioka et al., 2006; Degé and Schwarzer, 2017; Guo et al., 2018). Furthermore, evidence for a dose–response relationship between musical practice and working memory supports the view that training, rather than any predisposition, produces changes in working memory capacity (Bergman Nutley et al., 2014). However, associations between working memory capacity and measures of musical aptitude (Strait et al., 2011; Degé et al., 2015; Christiner and Reiterer, 2018; Ireland et al., 2018) suggest that training-related advantages are not independent of innate abilities. Thus the evidence presented here support the view that musical training and musical aptitude both contribute to working memory performance among children.

### Working Memory and Neural Oscillations in Children

As indicated previously in this review, working memory is associated with neural oscillations in adults, and this relationship has also been reported in children (Table 3). The majority of research on neural oscillations in children has used the delayed-match-to-sample test to measure oscillations during memory maintenance. As with work in adults, alpha oscillations have been implicated in working memory maintenance in children. For example, one study of 10–13-year-olds, in which alpha activity was measured during working memory maintenance showed that the lateralization of alpha power changed with the number of items in working memory (Sander et al., 2012). Specifically, when the number of items increased from low to medium, alpha power became more lateralized, whereas when the number of items increased from medium to high, alpha power became less lateralized. In contrast to the pattern seen in children, the adults showed an increase in alpha power lateralization as the number of items in working memory increased. In another study, Sato et al. (2018) reported higher alpha phase synchrony in 6-year-olds during the retention period of a delayed match to sample task for correct trials than for incorrect trials. Taken together, these reports indicate that the alpha network is engaged in memory maintenance during childhood.

**TABLE 3:** This table shows the results of studies on development, working memory, and oscillatory activity.

**Study**	**Task**	**FB**	**Behavioral results**		**Oscillatory results**				
				
				**Resting state**	**Pre-stimulus**	**Encoding**	**Maintenance**	**Retrieval**	**Load**
Machinskaya and Kurgansky, 2012	vDMS	αθβ	–	–	**θ** (*FM-T*): A > Chil. **β** (*F-C*): A > Chil.	–	**θ** (*T-P*): A < Chil. **α** (*F-P*): A > Chil.	–	–
Sander et al., 2012	vDMS	α	YA > Chil. (low load)	–	–	–	–	**α**: YA < Chil.	**High load:** **α**: YA > Chil. **Low load:** **α**: YA < Chil.
Rodriguez-Martinez et al., 2013	WMTB-C	θ	↑Age →↑WM	↑Age, WM →↓**θ**	–	–	–	–	–
Sato et al., 2018	vDMS	α	Accuracy = 80.15%	–	–	–	↑**α** (whole brain connectivity during correct trials)	–	–
Walker et al., 2019	vDMS	γ	-	–	–	–	**γ**: Ado. > Chil.	–	–

*YA, young adults; OA, adults; Ado, adolescents; Chil, children; F, frontal; C, central; T, temporal; P, parietal; O, occipital; M, medial; L, left; R, right.*

In contrast to the view that alpha oscillations are important for memory maintenance in children, another study showed no difference in alpha coherence, but an increase in theta coherence, during maintenance among 7–8-year-old children (Machinskaya and Kurgansky, 2012). Notably, adults in the latter study showed an increase in alpha coherence, but no change in theta coherence during memory maintenance. Rodriguez-Martinez et al. (2013) also showed that theta oscillations play a role in working memory in children, and that this role diminishes throughout development. Specifically, the authors showed correlations between resting state theta oscillations and composite scores on a nine-subtest measure of working memory, conducted across an age range of 6–26 years. Power spectral density of the theta range negatively correlated with age across the sample, and this relationship, when included in a bivariate model with reaction time scores on the oddball task, accounted for 90% of variability in working memory due to age (Rodriguez-Martinez et al., 2013).

Taken together, these results indicate that alpha and theta oscillations are involved in working memory during childhood, and that oscillatory patterns during working memory processing differ between children and adults. Alpha peak frequency (APF) increases from infancy through adulthood (Klimesch, 1999), and may be a useful marker of brain maturation (Valdés et al., 1990), and cognitive development (Mierau et al., 2016). However, due to divergent findings on the role of alpha (Sander et al., 2012; Sato et al., 2018) and theta (Machinskaya and Kurgansky, 2012) during working memory maintenance, further studies are needed to understand the contributions of different oscillations during working memory maintenance.

It is also possible that gamma rhythms play an important role in working memory maintenance, specifically in regard to its maturation across development. In a recent study, 10–12-year-old children were compared with 15–17-year-old adolescents on the delayed match to sample task. The older children showed increased gamma power during the delay phase, and an increased gamma response to transcranial magnetic stimulation (TMS) applied to the prefrontal cortex. It is worth noting that the TMS-elicited gamma power was positively correlated with working memory capacity (Walker et al., 2019).

Overall, the present discussion shows the utility of measuring oscillations for investigating the neural mechanisms of working memory processing at different stages of development. Because working memory capacity and duration improve over the course of development, and oscillatory patterns for the same abilities vary at different stages of development, studying how oscillations change throughout development could provide insights into the neural mechanisms of working memory. No study, to our knowledge, has been conducted in children to investigate relationships between music training, working memory, and neural oscillations. Thus, a significant gap in our knowledge is elucidation of how music training interventions and measures of musical aptitude might enhance or alter oscillatory activity throughout development.

## Relationship Between Music Training and Working Memory in Older Adults

In the sections above, we reported that adults with music training outperformed those without music training on several behavioral tasks of working memory. Consistent with those findings, older adults with music training also outperform older adults without music training. For example, Parbery-Clark et al. (2011) compared older musicians and non-musicians on auditory and visual working memory, and the ability to perceive speech in noise. They reported that musicians were significantly better at perceiving speech in noise and performed better in auditory, but not visuospatial, working memory capacity tasks. The study also revealed a linear relationship between auditory working memory and speech in noise performance, suggesting that these two functions are related. Grassi et al. (2018) also reported that older adult musicians outperformed older adult non-musicians on auditory and visuospatial working memory tasks, as well as auditory discrimination, but the groups did not differ on tests of short-term memory. In addition, Amer et al. (2013) reported that older adult musicians outperformed older adult non-musicians on several tests of executive functions, including visuospatial working memory. Taken together, these results reveal similarities and differences between adults and older adults with regard to relationships between music training and working memory. As with young adults, the benefits of music experience on working memory among older adults are not confined to the auditory domain. However, the finding that older musicians perform better than non-musicians on visuospatial working memory are in contrast to reports that young adult musicians and non-musicians do not differ on visuospatial working memory (Hansen et al., 2013). Also, life-long music training in older adults is not always associated with stronger working memory, as Hanna-Pladdy and MacKay (2011) reported strong correlations between music training and other executive functions, including cognitive flexibility, but not working memory. Some of the discrepancies in findings between young and older adults may be due to the effects of music training to compensate for age-associated decline in some executive functions.

Recently several studies have tested the potential of music intervention programs in reducing the deleterious effects of aging on cognition (Bugos et al., 2007; Hars et al., 2013; De Oliveira et al., 2014). In a study conducted by Bugos et al. (2007), older adults participated in individualized piano instruction consisting of motor dexterity exercises and learning music theory. Participants had lessons each week for 6 months and were tested on cognitive and working memory measures across three time points: pre-training, post-training, and following a delay period of 3 months. The experimental group obtained significantly higher scores post-training on the Trail Making Test and Digit Symbols than the untrained controls, indicating an improvement in visoscanning, perceptual speed, and working memory.

### Effects of Music Training and Interventions on Dementia

In addition to the benefits of music training on working memory in older adults, music training across the lifespan may serve as a protective factor against dementia (Verghese et al., 2003; Balbag et al., 2014). In a population based twin study, Balbag et al. (2014) showed older twins who played an instrument were 64% less likely to develop dementia than their co-twins. Contrary to these findings, a recent study (Kuusi et al., 2019) tracked the causes of death of classical musicians in Finland and found that musicians were just as likely to suffer from a neurodegenerative disorder as the general population. Taken together, these discrepant findings suggest that further studies are needed to determine whether music training is protective against neurodegenerative disorders.

Some studies have shown improvements in cognition and working memory in patients with dementia after active singing interventions (Särkämö et al., 2014; Maguire et al., 2015; Pongan et al., 2017). In a randomized controlled study, Särkämö et al. (2014) compared the effects of three different interventions on working memory on a group of patients with dementia. Each participant was assigned to one of three 10-week group-based interventions: singing, listening to music, or a usual care control group with physical or social activities. They reported that participants in the singing group showed a temporary improvement in working memory as measured by backward digit span. In contrast, Narme et al. (2014) found no cognitive improvements in patients with dementia after a 4-week music intervention. The discrepant results may be due to differences in intensity or duration of the interventions, suggesting that important parameters for interventions, such as the effective “dose” of training, are not yet well established.

Overall, these studies show that music training may protect against age-related decline in working memory, as well as improve performance among older adults who show decline in working memory. Importantly, music training may also be useful in the prevention and treatment of dementia.

### Neural Oscillations in Older Adults

In comparison with young adults, the neural oscillations of older adults undergo several changes. As described in the paragraphs that follow, these changes may be related to age-related decline in working memory and cognition. However, there is a significant gap in our knowledge of the extent to which music training and music interventions can prevent or restore patterns of oscillatory activity and working memory among older adults. Some of the most often reported age-related changes in brain wave activity are in: (1) activity during resting state recording (slowing of alpha frequency and theta changes); (2) changes in functional connectivity, or coherence; and (3) changes in power (or ERS/ERD) across different frequency bands and electrode sites during active recording. We discuss age-related changes in each of these measures below.

#### Resting State Alpha Frequency Slows Among Older Adults

Some of the most well studied age-related changes occur during resting-state recordings. Resting state alpha frequency increases throughout development (Lindsley, 1939; Stroganova et al., 1999; Marshall et al., 2002) reaching a peak in young adulthood (Chiang et al., 2011) and then slowing in older adults. The slowing of dominant alpha in older adults is a well-known phenomenon that may indicate various cognitive deficits (Obrist, 1954; Duffy et al., 1984; Köpruner et al., 1984; Aurlien et al., 2004; Chiang et al., 2011; Scally et al., 2018). Clark et al. (2004) found that age-related slowing of alpha, as measured by APF, was negatively correlated with working memory capacity. In the elderly, APF slowing is more dramatic in anterior than in posterior recording sites compared to young adults. In addition, variability in APF is negatively correlated with working memory across the lifespan, indicating the effectiveness of APF as a biomarker for working memory, a finding that has been replicated (Grandy et al., 2013). It is possible that normalizing the APF could restore some age-related decline in cognition. Training older individuals to increase APF through neurofeedback may improve processing speed, inhibitory control, and working memory (Angelakis et al., 2007).

Alpha peak frequency could also serve as a potential biomarker for mild cognitive impairment (MCI) (Babiloni et al., 2018) as it is lower for the MCI population than for normal older adults, as well as being correlated with MMSE scores. Furthermore, APF in posterior sites is positively correlated with hippocampal volume, which is reduced in the mild cognitively impaired population (Garcés et al., 2013). Likewise, a negative correlation has been found between frontal theta power and hippocampal volume (Grunwald et al., 2001). Overall, studies seeking to investigate the effects of music training on cognition and working memory in older adults should consider the relationship between aging, cognition, and changes in oscillatory activity during resting state, like APF.

Recording of neural oscillations during working memory may elucidate neural changes associated with aging (Table 4). Young and elderly adults show different patterns of ERS/ERD in alpha (Sander et al., 2012) and theta (Karrasch et al., 2004) during active recording in working memory tasks, even though the two groups may perform similarly. Older adults also tend to show lower theta power during working memory maintenance (Cummins and Finnigan, 2007; Kardos et al., 2014, Tóth et al., 2014) in comparison with young adults. Older adults also have lower theta (Kardos et al., 2014) and alpha power (McEvoy et al., 2001; Sander et al., 2012) in high working memory load tasks. Moreover, Sander et al. (2012) reported that older adults and children demonstrate similar activity during working memory tasks. On average, both older adults and children have higher alpha power in comparison to young adults for low working memory loads. In children, higher alpha power is likely due to less well-developed neural mechanisms of working memory. In contrast, higher alpha power, and lower theta, in older adults is likely due to compensatory mechanisms.

**TABLE 4:** This table shows the results of studies on aging, working memory, and oscillatory activity.

**Study**	**Task**	**FB**	**Behavioral results**	**Oscillatory results**			
				
				**Encoding**	**Maintenance**	**Retrieval**	**Load**
Cummins and Finnigan, 2007	Verbal Sternberg	θ	YA = OA	–	**θ**(*F, C*): YA > OA	**θ**(*F, C*): YA > OA	–
Kardos et al., 2014	vDMS	θ	YA > OA	–	**θ** (*P, O*): YA > OA	–	**High load:** **θ** (*P, O*): YA > OA
Karrasch et al., 2004	Auditory Sternberg	αβθ	YA = OA	**θ**(*P*): YA > OA **α**(*RT*): YA < OA **β**(*C*): YA < OA	–	**α** (*C, T, O*): YA < OA **β** (*C, T, O*): YA < OA **θ** (*O*): YA > OA	–
McEvoy et al., 2001	Spatial working memory	αθ	YA = OA	–	–	–	**High load:** **θ** (*FM*): YA > OA **α**: YA > OA
Sander et al., 2012	vDMS	α	YA > OA (low load)	–	–	**α**: YA < OA	**High load:** **α**: YA > OA **Low load:** **α**: YA < OA
Tóth et al., 2014	vDMS	θ	YA > OA	–	**θ**: YA > OA	–	–

*YA, young adults; OA, older adults; F, frontal; C, central; T, temporal; P, parietal; O, occipital; M, medial; L, left; R, right.*

#### Connectivity Decreases Among Older Adults

Age-related reductions of interhemispheric coherence have been observed in the delta, theta, alpha, and beta oscillations (Duffy et al., 1996; Kikuchi et al., 2000) during resting state recording. Global connectivity is also affected with age. Global alpha connectivity during resting state decreases with old age (Scally et al., 2018). Testing a large sample of 17,722, Vysata et al. (2014) reported an age-related decrease in global theta and alpha coherence. In a longitudinal study Fjell et al. (2016) found that reduction in connectivity is related to decline in inhibitory control. While there are not many studies that have explored the relationship between age-related declines in functional connectivity and working memory, it has been shown that working memory may improve by restoring theta synchronization in frontotemporal regions through transcranial alternating current stimulation (tACS; Reinhart and Nguyen, 2019). As mentioned previously, young adult musicians show higher theta synchrony during working memory tasks as well as better performance in those tasks (Cheung et al., 2017; Dittinger et al., 2017). Improved coherence as a result of music training may underlie the cognitive and working memory benefits seen in older musicians. Music training may help in strengthening some connections that may become weaker due to aging.

Consistent with the findings reported for young adults, older adults with music training outperform older adults without music training on working memory tasks. These findings indicate that cognitive benefits that occur as a result of music training persist throughout the lifespan. Musical interventions in older non-musicians may also help in ameliorating age-related cognitive decline. Both non-pathological and pathological aging are accompanied by disruptions in brain wave activity, and these disruptions may also reflect working memory decline. While there are no studies showing the effects of music training on oscillatory activity in older adults, studies in young adults show the potential of music training to alter neural oscillations in ways consistent with enhanced working memory. It remains to be determined whether or not music training may improve some of the functional connectivity that is lost during aging. Studies with mice have shown that entrainment of oscillations in the gamma range through visual (Iaccarino et al., 2016) and auditory (Martorell et al., 2019) stimulation improves spatial and recognition memory and reduces the neuropathology associated with Alzheimer’s disease. The entrainment of neural oscillations is taken up in the following section.

## Entrainment of Neural Oscillations

As discussed throughout this review, music training may alter oscillatory activity and functional networks associated with working memory. These findings suggest that oscillations may be targeted selectively to modulate working memory performance. Some studies have used stimulation techniques to disrupt oscillatory activity underlying WM processes; however, the effects on behavior may be at the individual level. For example, when repetitive TMS is applied over parietal brain regions during memory delays, alpha-tuned stimulation impairs performance for individuals with high baseline WM capacity (Li et al., 2017), or for those who show increased alpha power during the delay period (Hamidi et al., 2009). Transcranial electrical stimulation applied over frontal and parietal regions during consecutive days of WM training has negative effects on WM performance and resting state connectivity (Möller et al., 2017). These findings seem to suggest a causal link between oscillatory rhythms and WM, such that perturbation of these rhythms, particularly during memory delays, negatively impacts maintenance of information over time.

If disruption of oscillatory activity impairs WM processes, can entrainment of specific rhythms enhance behavior? Indeed, a recent study using TMS examined whether tuning TMS to a specific frequency could enhance entrainment of neural networks associated with working memory (Albouy et al., 2017). TMS tuned to theta frequency enhanced performance during an auditory memory manipulation task; furthermore, theta power in correct trials positively correlated with musical experience. Similarly, theta-tuned tACS improved performance on an *N*-back task (Pahor and Jaušovec, 2018).

Stimulation techniques such as TMS and tACS, while non-invasive, require specialized equipment and expert administration. However studies have shown that simply listening to or tapping along with the rhythmic structure of music entrains the brain’s low-frequency oscillations (Lakatos et al., 2008; Besle et al., 2011). For example, passive listening to musical sequences induces changes within alpha (Bridwell et al., 2017) and increases coupling between delta and high beta frequency ranges (Adamos et al., 2018). Word lists that are sung rather than spoken increase alpha coherence in bilateral frontal areas (Thaut et al., 2005) known to support learning-related processes (Sato et al., 2018). Furthermore, enhanced coherence to rhythmic musical stimuli correlates with improved memory for subsequent speech stimuli (Falk et al., 2017).

Tapping along with music rhythms induces beat-related entrainment (Nozaradan et al., 2015) as well as changes in functional somatosensory networks (Daly et al., 2014) and behavior (Nozaradan et al., 2016; Crasta et al., 2018). Importantly, rhythm entrainment is not limited to the auditory modality (Okawa et al., 2017) and can occur in the absence of an external stimulus. For example, entrained oscillatory activity persists during brief pauses in a rhythmic pattern (Stupacher et al., 2016), and varies not only with rhythm-based predictions of directly observed sequences, but also with memory-based predictions of imagined sequences (Breska and Deouell, 2017; Okawa et al., 2017). As discussed previously in this review, musicians show enhanced oscillatory activity and coherence during working memory delays, which may facilitate improved music-related error processing and pattern predictions shown in musicians compared to non-musicians (Doelling and Poeppel, 2015; Stupacher et al., 2017; Harding et al., 2019).

In addition to rhythm entrainment, oscillatory activity associated with working memory may be targeted by presenting two tones of different frequencies, one frequency to each ear; the resulting percept is of a *binaural beat* equal to the difference between the two frequencies (Licklider et al., 1950). Recent studies show that listening to binaural beats induces changes in cortical networks associated with information processing. For example, increased gamma and beta power over frontal and central regions in response to binaural beats improved short-term memory of middle-list items on a serial recall task (Jirakittayakorn and Wongsawat, 2017). In addition, accuracy on visuospatial (Beauchene et al., 2016) and verbal *N*-back (Beauchene et al., 2017) working memory tasks increased in response to binaural beats, which were thought to strengthen cortical networks involved in maintenance and retrieval of task-related information. Even passive listening to binaural beats induced changes in alpha power over frontal, temporal, and parietal lobes, with greater power increases in participants with musical experience (Ioannou et al., 2015). Thus, binaural beat stimulation may be a promising tool to entrain cortical networks involved in working memory.

## Summary and Future Directions

Music training requires storage, manipulation, and integration of complex pitch and temporal sequences. In this way, it shares several features with commonly used measures of working memory. Therefore, it is not surprising that music training is related to enhancements in executive functions, including working memory. This review provides evidence that the study of neural oscillations is important for understanding the neural mechanisms underlying relationships between music training and working memory. In specific, we provide evidence that measurement of neural oscillations is particularly useful for studying temporal stages of memory and cognition that may occur over spans of seconds to minutes. In addition, we show that a lifespan approach to the study of relationships between music training, working memory, and neural oscillations reveals similarities and differences in working memory and underlying neural events at different stages of life. The following are some of the important specific points raised in this review, as well as suggestions for further investigation.

First, behavioral studies in adults show that benefits of music training are not restricted to auditory working memory, but may extend to the visual or other sensory modalities. In addition, music training may influence working memory capacity more selectively than working memory duration.

Second, music training is related to distinct patterns of modulation of oscillations that are related to encoding, maintenance, and retrieval phases of working memory in adults. Table 1 lists studies in which measures of neural oscillations were used to: (1) dissociate temporal components of working memory such as encoding, maintenance, and retrieval; (2) investigate working memory processing demands, such as changes in memory load and inhibition, and (3) measure short and long-range synchronous activity to examine the relative contributions of distributed brain regions involved during working memory tasks. Table 2 lists studies that examine the degree to which music training influences oscillatory activity during working memory. While we have discussed differences in activity within frequency bands related to musical expertise, to our knowledge there are no reports of whether cross-frequency coupling during working memory varies as a function of music training. Thus while music training-dependent interactions between frequency bands remain to be investigated, there is evidence that music training may alter neural connectivity in functional networks underlying working memory.

Third, neural oscillations that support working memory and related cognitive processes change across the lifespan, and may serve as targets for music training or selective entrainment. Investigators have not yet studied relationships between music training and neural oscillations in children or older adults. Thus it remains to be determined whether or not relationships between music training and neural oscillations observed in adults will be the same as those observed in children or older adults. It is notable that alpha and theta oscillations are involved in working memory during childhood, and that oscillatory patterns during working memory processing differ between children and adults (Table 3). In addition, theta coherence declines with age but is enhanced during working memory maintenance in young musicians compared to non-musicians. Alpha activity, which is associated with inhibition in young adults, is also shown to decline with age, as does inhibitory processing. Accordingly, selective modulation of theta and alpha may show age-related changes in working memory maintenance and distractor suppression, respectively (Table 4). Future studies could test this hypothesis by comparing the effects of music training or targeted entrainment on theta maintenance or alpha inhibition in young and older adults.

Finally, recent findings that entrainment of neural oscillations can enhance memory and cognition, and may reverse markers of neuropathology in models of Alzheimer’s disease, suggest that further study of the role of neural oscillations in these processes will be necessary to guide development of therapeutic interventions for enhancement of cognition and for treatment of neural disorders across the lifespan. Of importance, music training, or targeted applications of musical stimuli, may serve as natural and non-invasive interventions for altering or entraining neural oscillations.

## Author Contributions

All authors contributed to the conception, organization, drafting, and revision of this manuscript.

## Conflict of Interest

The authors declare that the research was conducted in the absence of any commercial or financial relationships that could be construed as a potential conflict of interest.
